# NIR-II-absorbing diimmonium polymer agent achieves excellent photothermal therapy with induction of tumor immunogenic cell death

**DOI:** 10.1186/s12951-023-01882-7

**Published:** 2023-04-20

**Authors:** Han Xu, Huaping Deng, Xiaoqian Ma, Yushuo Feng, Ruizhen Jia, Yiru Wang, Yaqing Liu, Wenli Li, Shanshan Meng, Hongmin Chen

**Affiliations:** 1 State Key Laboratory of Molecular Vaccinology and Molecular, Diagnostics & Center for Molecular Imaging and Translational Medicine, School of Public Health, Xiamen University, Xiamen, 361102 China; 2 State Key Laboratory of Organic Electronics and Information Displays & Institute of Advanced Materials (IAM), Jiangsu Key Laboratory for Biosensors, Nanjing University of Posts & Telecommunications, Nanjing, 210023 People’s Republic of China

**Keywords:** NIR-II absorbing organic agent, Diimmonium salt, Photothermal therapy, Tumor immunogenic cell death

## Abstract

Photothermal therapy has shown great promise for cancer treatment and second near-infrared (NIR-II) -absorbing particles could further improve its precision and applicability due to its superior penetration depth and new imaging ability. Herein, high NIR-II-absorbing polymer particles were prepared by using soluble isobutyl-substituted diammonium borates (P-IDI). The P-IDI showed stronger absorption at 1000–1100 nm, which exhibited excellent photostability, strong photoacoustic imaging ability and high photothermal conversion efficiency (34.7%). The investigations in vitro and in vivo demonstrated that the excellent photothermal effect facilitated complete tumor ablation and also triggered immunogenic cell death in activation of the immune response. The high solubility and excellent photothermal conversion ability demonstrated that polymer IDI particles were promising theranostic agents for treatment of tumors with minor side effects.

**Supplementary Information:**

The online version contains supplementary material available at 10.1186/s12951-023-01882-7.

## Introduction

Photothermal therapy (PTT) is a new paradigm towards cancer therapy due to the advantages of noninvasiveness, localized treatment and irradiation with spatiotemporal selectivity. In its design, PTT employed photothermal agents absorbing optical energy convert into heat power and accumulated in the tumor tissue to kill tumor cells [1, 2]. Gold shells have been employed in clinical trial for photothermal tumor therapy by using 808 nm laser. However, the application of NIR-assisted therapeutic methods remains challenging in treating deep tumors due to low tissue penetration of light [3]. It remains to be challenging for deep-seated cancer management.

Efforts have been made for exploitation of the photothermal agents in the second NIR (NIR-II, 1000−1700 nm) window, owing to the intrinsic advantages of the lesser photo scattering and preferable penetration depth [4–6]. Inorganic photothermal materials, such as gold nanorods, or gold nanoshells (GNS) with high absorption in the NIR-II region [7, 8]. Nevertheless, inorganic materials are difficult to degrade in the body, it would hamper their further application. By contrast, organic photothermal materials have captured great attentions attributed to their biodegradable and metabolizable advantages. The organic dyes, such as diammonium salts (DIs) [9], squaryliums [10] and cyanines [11] with strong NIR-II absorption are good candidates for photothermal material applications. However, they possess poor thermal stability. It is surprising that DIs exhibit thermal stability through modification as isobutyl-substituted diammonium borate (IDI). Molecular imaging technologies endow in vivo cancer therapy with great precision and safety. In particular, photothermal agents exhibit high NIR optical absorption, have been appropriate candidates for photoacoustic imaging (PAI). As an emerging optical imaging strategy, PAI is based on photoacoustic effect, through sensing acoustic pressure waves produced from absorbed photon energy. PAI enables high-resolution visualization of the biological tissues for tumor detection and therapeutic monitoring.

The development of tumor depends on the complex tumor microenvironment (TME), in which includes myeloid-derived suppressor cells (MDSCs) and the regulatory T cells (Tregs) restrain the activation of effector T cells and being the immunosuppressive state [12–16]. Consequently, tumors turn to be “cold” and insensitive to immune response [17–20]. Mild photothermal therapy is reported to reprogram the “cold” TME [21–26]. It has been demonstrated that PTT can cause immunogenic cell death (ICD) [27, 28], generating tumor-associated antigens (TAAs) and damage-associated molecular patterns (DAMPs), which could activate dendritic cells and stimulate an antigen presentation to T cells [12, 29–32]. However, currently most of the light-absorbed agents are in NIR-I window (700 − 900 nm) for imaging and phototherapy. It remains to be challenging for further biomedical application with poor penetration depth.

**Scheme 1.**
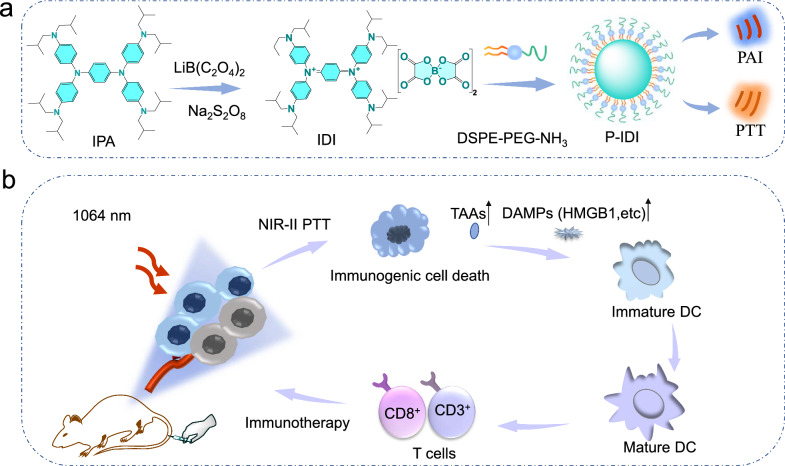
Schematic illustration of NIR-II-absorbing diimmonium polymer agent achieves excellent photothermal therapy with induction of tumor immunogenic cell death. **a** A scheme that illustrates the synthesis of P-IDI via nanoprecipitation. **b** Tumor immunogenic cell death induced by P-IDI based PTT

In this study, we designed a novel P-IDI nanoparticle through the oxidation of neutral amines and DSPE-PEG-NH_2_ modification strategy, which exhibited uniform size, superior biocompatibility, high NIR-II absorption, and excellent photothermal conversion efficiency (Scheme 1). The PAI and PTT effects of P-IDI were validated in a mouse 4T1 subcutaneous tumor model of breast cancer. As displayed by the PAI results, the nanoparticle displayed high-efficient tumor accumulation. In vivo PTT was also performed, in which we discovered the P-IDI showed a high efficiency tumor elimination effect under 1064 nm laser irradiation with 1 W/cm^2^. Besides, the effects of PTT induced ICD were also explored (Scheme 1). P-IDI should thus be a new category of theranostic agents for enhanced cancer diagnosis and treatment, which paves a new avenue for their potential clinical applications.

## Results and discussion

### Fabrication and characterization of P-IDI

**Fig. 1:**
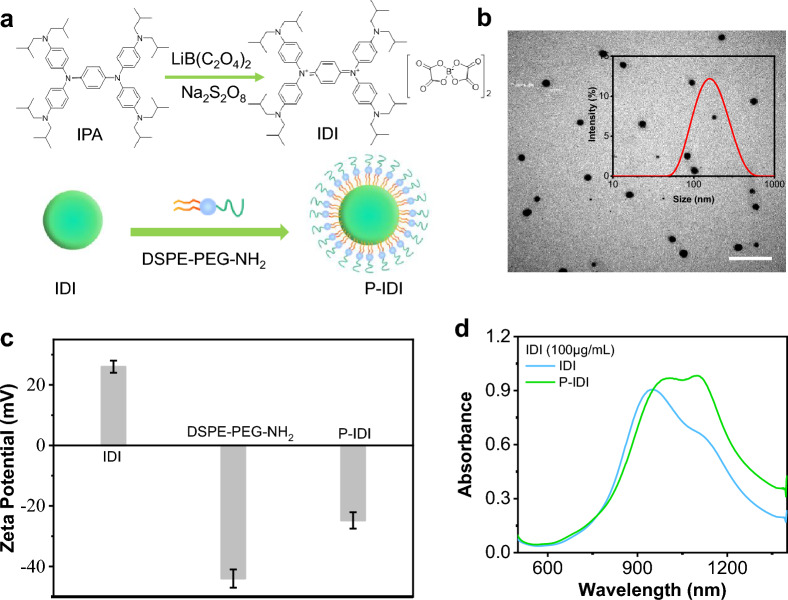
Preparation and characterization of P-IDI. **a** A scheme that illustrates the synthesis of P-IDI via nanoprecipitation. **b** TEM image of P-IDI, scale bar: 1 μm. Inset: DLS profile of P-IDI. **c** Zeta potentials of IDI, DSPE-PEG-NH_2_ and P-IDI. **d** The absorption spectra of IDI and P-IDI. The concentrations were normalized to IDI as 100 µg/mL

The soluble diammonium salts IDI were synthesized by oxidizing neutral amine IPA (N,N,N′,N′-Tetrakis[4-(diisobutylamino)phenyl]-1,4-phenylenediamine) in the presence of bis(oxalate)borate and persulfate with a yield of 89.2% [9, 33] (Figs. 1a**,** Additional file 1: Figure. S1-4). The structure of IDI and its derivatives have rarely been characterized thus far, mainly due to its paramagnetic properties [9]. The magnetic properties of IDI were measured using a physical property measurement system [34] (Additional file 1: Figure S5). To enhance their solubility, the IDI were encapsulated in DSPE-PEG-NH_2_ to form polymer nanoparticle P-IDI (Fig. 1a). The hydrated size of P-IDI was determined to be 146 ± 16 nm, which was slightly higher than that of sizes obtained from TEM image (123 ± 11 nm) (Fig. 1b). Zeta potentials changed from 26 ± 2.4 mV of IDI to -24.8 ± 2.7 mV of P-IDI (Fig. 1c). The stabilities of IDI and P-IDI were also evaluated, compared with IDI, P-IDI displayed satisfactory stability in PBS and FBS even for ten days (Additional file 1: Figure S6). Surprisingly, after encapsulating in DSPE-PEG-NH_2_, the absorbance peaks red-shifted from 948 nm with a should peak at 1100 nm of IDI to strong peaks in the range of 1000–1160 nm of P-IDI (Fig. 1d). The most important was that the absorbance was 0.29 at 808 nm, and 0.96 at 1064 nm (Fig. 1d). It is well-known that the second near-infrared (NIR) window takes advantage of the water transparency window, so it is able to penetrate deeper into biological substrates with reduced scattering [35]. The stronger absorption of P-IDI in the optical transmission window of oxygenated blood, deoxygenated blood, skin and fatty tissue makes it as an attractive NIR-II photo-stimulated diagnostic and/or therapeutic agent for tumor managements [36].

### In vitro photonic hyperthermia performance of P-IDI

**Fig. 2:**
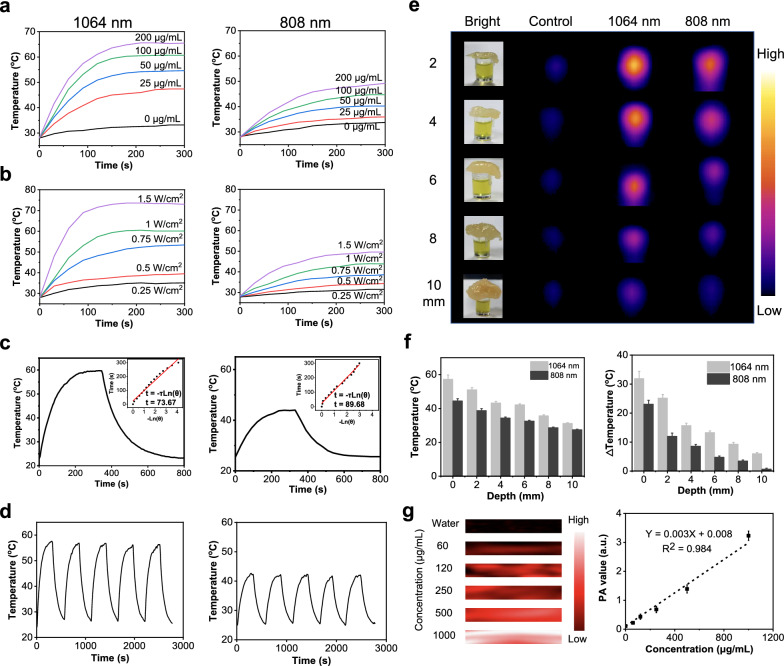
Photothermal and photoacoustic properties of P-IDI. **a** Photothermal conversion curves of P-IDI at concentrations 0–200 μg IDI/mL and laser irradiation (1 W/cm^2^) of 1064 nm and 808 nm. **b** Photothermal conversion curves of P-IDI (100 μg IDI/mL) under laser irradiation of 1064 nm and 808 nm (0.25–1.5 W/cm^2^). **c** Photothermal efficacy of P-IDI (100 μg IDI/mL) under 1064 nm and 808 nm laser irradiation. **d** Thermal stability of P-IDI (100 μg IDI/mL) upon 1064 nm (1 W/cm^2^) and 808 nm (1 W/cm^2^) laser irradiation. **e** Simulated deep-tissue penetration and photothermal efficacy of P-IDI (200 μg IDI/mL) using chicken breast under 1064 nm (1 W/cm^2^) and 808 nm (1 W/cm^2^) laser irradiation. **f** Temperature increase of P-IDI (200 μg IDI/mL) upon the laser irradiation from (**e**). **g** PA phantom images and PA amplitudes of P-IDI upon 808 nm laser irradiation

As P-IDI showed excellent NIR-II absorption, its photothermal property was investigated. The relationships on concentrations (0–200 μg IDI/mL), laser wavelengths (808 nm and 1064 nm) and power densities (0.25–1.5 W/cm^2^) were assessed. It was clearly demonstrated that the absorbance of 1064 nm was four-fold higher than that of 808 nm, all the photothermal properties under 1064 nm irradiation were better than that of 808 nm (Fig. 2a, b). Under all the same condition, the temperatures under 1064 nm irradiation could reach to as high as 70 °C, however, the temperatures under 808 nm irradiation were lower than 50 °C (Fig. 2a, b). Consequently, the photothermal conversion efficiency (η value) of P-IDI was calculated to be 34.7% at 1064 nm, which was 1.2-fold enhancement of 808 nm irradiation (29.8%) (Fig. 2c). Compared to the common NIR-II photoagents (ICG 3.1% [37], IR1048-MZ 20.2% [38], and Au NRs 21% [39]), its efficacy was significantly better than them. These findings directly proved that P-IDI could efficiently and rapidly convert photo energy to heat under laser irradiation. Photostability is another important evaluation parameter for a potential photothermal agent. After five cycles of heating and cooling processes, both irradiations at 1064 nm and 808 nm showed minor changes on each test recorded (Fig. 2d), indicating that P-IDI was a thermal-durable agent.

To assess the potential for deep-tissue imaging and therapy, simulation deep-tissues were investigated. Chicken breasts with different thicknesses (2, 4, 6, 8, and 10 mm) were employed (Bright panel, Fig. 2e**),** and lasers were irradiated up of the chicken breasts. The temperature was monitored by a thermal imager. As shown in Fig. 2i–k, 1064 nm showed better penetration to generate heat energy than that of 808 nm, even the thickness of chick breast is around 1 cm (Fig. 2f). More importantly, under laser irradiation, P-IDI generated strong photoacoustic signals with perfect linearity between the intensity of PA signal and the dose of P-IDI (Fig. 2g).

### In vitro photonic hyperthermia of P-IDI against cancer cells

**Fig. 3:**
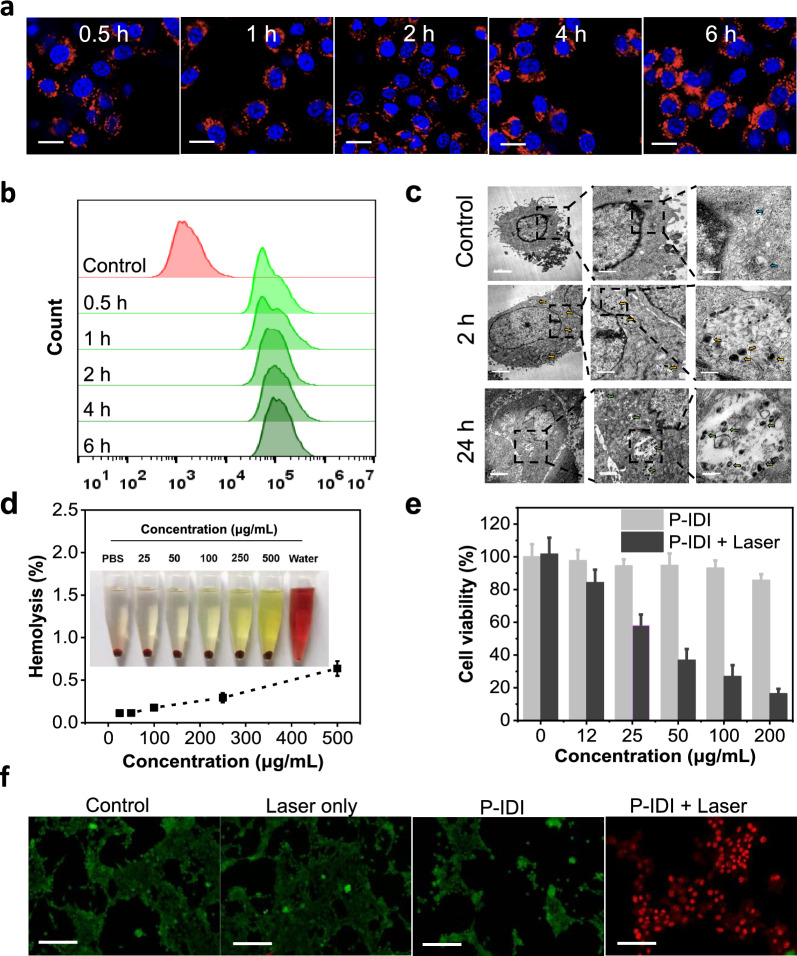
The efficiently induce tumor cell death by P-IDI. **a** Confocal images of the 4T1 cells after incubating with P-IDI (100 μg IDI/mL) for 0.5, 1, 2, 4, and 6 h (Blue: Hoechst 33342; Red: Cy5.5). Scale bar: 50 µm. **b** Flow cytometry analysis of internalization of P-IDI. **c** Representative bio-TEM images of 4T1 cells incubated with P-IDI (100 μg IDI/mL) for 2 and 24 h. Scale bars were 2 µm, 1 µm and 200 nm from left to right, respectively. **d** Hemolysis analysis of P-IDI (25–500 μg IDI/mL) solutions at various concentrations (mean ± SD, n = 3). The mixtures after kept standing for 3 h were centrifuged to detect the hemoglobin in the supernatant visually (inset). **e** Cell viabilities of 4T1 cells measured by MTT assays, after incubating with different concentration of P-IDI with or without 1064 nm laser (1 W/cm^2^, 5 min) irradiation. **f** Fluorescence images of Calcein AM (live cells, green fluorescence) and propidium iodide (PI) (dead cells, red fluorescence) co-stained 4T1 cells with different treatments, including control and P-IDI (100 μg IDI/mL) for 12 h with or without laser irradiation (1064 nm, 1 W/cm^2^, 5 min). Scale bar: 100 µm

Confocal laser scanning microscopy (CLSM) imaging (Fig. 3a) and flow cytometry analysis using cyanine 5.5 (Cy5.5) -labeled P-IDI (Fig. 3b) showed that P-IDI were efficiently internalized into murine breast carcinoma cells lines 4T1 cell lines. After incubation for only 0.5 h, clear red signals and efficient uptake were observed in cells (Fig. 3a, b). The cell uptake and biodegradability of P-IDI were investigated by bio-TEM (Fig. 3c). After incubation for 2 h, P-IDI were obviously taken in by 4T1 cells, and most of which were obviously present in lysosomes. The morphology of the P-IDI was spherical within 2 h after being ingested by the cells, while gradually degraded after 24 h, and the volume also decreased accordingly. Morphological change of the nanospheres testified an obviously time-dependent biodegradation of P-IDI. These results suggested that the P-IDI could be gradually biodegraded in a time-dependent manner. Subsequently, P-IDI did not cause obvious hemolysis after incubation with murine erythrocyte (Fig. 3d), which ensured its suitability for further biological applications. And in vitro cytotoxicity of P-IDI evaluated by standard MTT assay showed that after incubation with P-IDI (0–200 μg IDI/mL) for 24 h, there was no significant toxicity (Fig. 3e). Laser irradiation induced dramatically decrease of cell viability and the decrease ratio was mainly relied on its concentrations and laser powers (Figs. 3e**,** Additional file 1: Figure. **S7**). In the presence of laser irradiation, when the concentration of P-IDI was higher 50 μg IDI/mL, the cellular viability decreased to lower than 36.9 ± 6.7% (Fig. 3e). And, when the concentration of P-IDI was locked as 100 μg IDI/mL, laser power was set as higher than 0.5 W/cm^2^, the cellular viability decreased to lower than 50% (Additional file 1: Figure S7). Live/dead assay also showed that at the power of 0.5–1.5 W/cm^2^, almost no green signals (live cells) were observed, in contrast, bright and intense red signals (dead cells) were evidently observed (Additional file 1: Figure S8). In vivo thermal imaging also confirmed that the heating efficiency of P-IDI enriched in subcutaneous tumor of mice was positively correlated with laser power (Additional file 1: Figure S9). So, in the following investigations, the concentration and laser power were set as 100 μg IDI/mL and 1 W/cm^2^ to achieve better cancer management efficacy. Under the setting condition, only P-IDI in the present of laser showed high-density red fluorescent signals, indicating effectively NIR-II photothermal efficacy of cancer cells (Fig. 3f).

### Biocompatibility and biodistribution of P-IDI in mice

Above results demonstrated completely the excellent photothermal efficacy in NIR-II region, its compatibility was then investigated in normal mice. After injecting P-IDI (10 mg IDI/kg) by tail vein, blood was extracted for serum chemistry analysis, kidney and renal functions. No significant changes were caused in mice (Additional file 1: Table S1, S2).

**Fig. 4:**
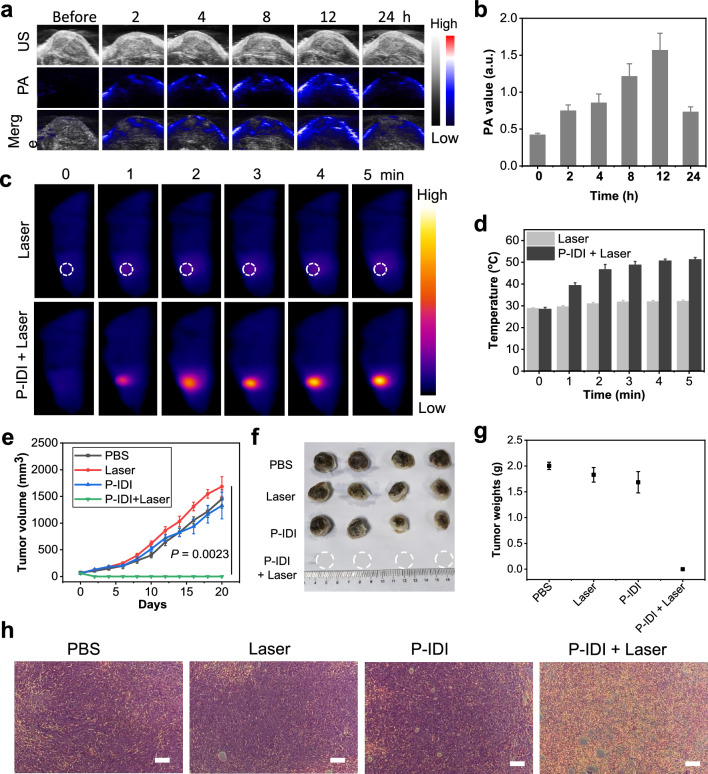
The distribution and therapeutic efficacy of P-IDI on 4T1 Tumor-bearing Mice. **a** 2D ultrasonic (US), PA and merge images of tumor tissues from pre- and post-injection of P-IDI (10 mg IDI/kg). **b** The corresponding PA amplitude of tumor tissues from (a). **c** Thermal images of 4T1 tumor-bearing mice with different treatments. **d** Temperature of 4T1 tumors upon laser irradiation as a function of irradiation time (1064 nm, 1 W/cm^2^). **e** Tumor growth curves of 4T1 tumor-bearing mice after various treatments (n = 4). **f** Images of representative tumors taken from mice in different formulations after the 21-day treatment period. **g** Final tumor weights of 4T1 tumor-bearing mice exposed to different formulations after different treatment. **h** H&E staining images of tumor sections harvested from 4T1 tumor-bearing mice (PBS, Laser only, P-IDI, P-IDI + Laser) after treatments. Scale bar: 100 µm. Statistical significance was assessed via unpaired two-sided Student t-test. *P < 0.05, **P < 0.01, ***P < 0.001, ****P < 0.0001 versus control

Effective accumulation in tumors is important for the further application of the diagnostic and therapeutic of tumors. Firstly, the in vivo PA imaging performance of P-IDI was investigated in subcutaneous tumors by monitoring the signals at tumor regions after tail vein injection. The results showed that the gradually increased PA signals in tumor regions and reached the peak at 12 h post-injection time point (Fig. 4b). To double confirm the accumulation, a NIR dye Cy5.5 was loaded on P-IDI. After 12 h administration, the tumor area showed strong fluorescence signal (Additional file 1: Figure S10), and the corresponding signal was also found in the liver, indicating that the nanoparticle was mainly enriched in the liver and tumor area of mice.

### In vivo PTT evaluation based on P-IDI

After confirming the highest accumulation time point, the photothermal conversion efficacy in vivo was assessed using subcutaneous 4T1 tumor-bearing model. Tumors were irradiated by 1064 nm laser (1 W/cm^2^) for 5 min at 12 h post-injection time point. The temperature in the tumor regions that monitored by an IR thermal camera quickly increased to 46.7 °C in mice administrated with P-IDI in the present of laser during the first 2 min and then maintained at 52.3 °C within 5 min (Fig. 4c). In comparison, the temperature of the tumors in the PBS group in the present of laser irradiation showed a tiny increase (Fig. 4d), demonstrating that P-IDI was capable of significantly elevating the local tumor temperature upon the irradiation in the NIR-II window. After irradiation, the tumor volumes of mice received various treatments were monitored to evaluate the in vivo photothermal ablation efficacy (Fig. 4e–g). P-IDI-mediated NIR-II PTT achieved complete tumor elimination with the rate close to 100% after 2-Day treatment, and no tumor regrowth occurred during the observation period of 21 days (Fig. 4e). At the end day of treatments, the tumors of control groups or the tissues in the scar regions of the PTT group were extracted to weight and for further pathology analysis. The photographs and the weights of tumors validated P-IDI could remarkably suppress tumor growth under the laser irradiation (Fig. 4f, g). Representative hematoxylin & eosin (H&E) images of tumor tissues after different treatments showed obvious tumor cells in control groups and normal cells in PTT group (Fig. 4h). In addition, the body weights of tumor-bearing mice were not influenced by these treatments, and H&E staining of the main organs of the mice exhibited no obvious signal of tissue damage or inflammation lesion in all treatment groups suggesting that photothermal treatment induced by P-IDI featured high therapeutic efficacy and excellent biocompatibility (Additional file 1: Figures S11, S12). These findings confirmed that P-IDI could serve as an efficient photothermal agent with excellent biocompatibility.

### ICD in vivo induced by PTT

**Fig. 5:**
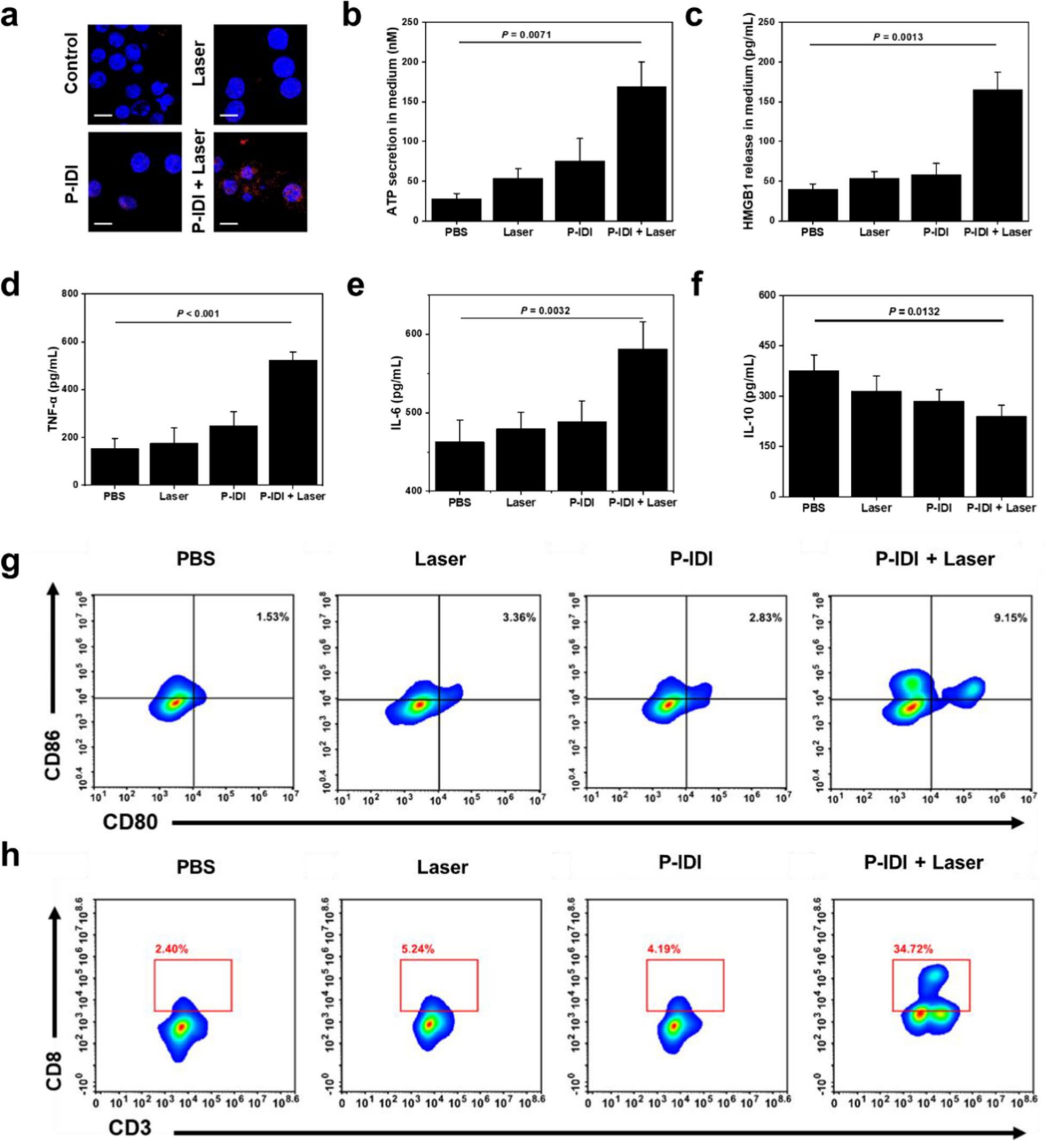
ICD induced by P-IDI based PTT. **a** Representative fluorescence images of 4T1 cells showing CRT exposure on the surface after various treatment (100 μg IDI/mL, Laser: 1064 nm, 1 W/cm^2^ for 5 min). Scale bar: 10 μm. **b** ATP release after various treatments. **c** HMGB1 secretion in the medium after various treatments. **d**–**f** TNF-α, IL-6, IL-10 of DCs after incubation with 4T1 cells treated by P-IDI (100 μg IDI/mL) with laser irradiation (1 W/cm^2^, 5 min). **g**, **h** Flow cytometric analyses of the populations of matured DC cells and CD8^+^ T cells in splenocytes of mice immunized after the different treatment. Statistical significance was assessed via unpaired two-sided Student t-test. *P < 0.05, **P < 0.01, ***P < 0.001, ****P < 0.0001 versus control

Previous studies have revealed that PTT can lead to immunogenic cell death (ICD) in tumor cells. This is because PTT not only causes tumor cell death, but also releases tumor antigens and endogenous adjuvants [40]. These endogenous adjuvants, such as calreticulin (CRT), high mobility group box 1 (HMGB1), and adenosine triphosphate (ATP), could increase tumor immunogenicity [41]. In the present, after PTT treatment (100 μg IDI/mL, laser irradiation: 1064 nm, 1 W/cm^2^ for 5 min), surface-exposure of CRT (Fig. 5a), secretion of ATP (Fig. 5b), and HMGB1 (Fig. 5c) of tumor cells were evaluated by immune-fluorescence and enzyme-linked immune sorbent assay (ELISA), respectively. Compared to control group, strong red fluorescence of CRT was observed in cells treated with PTT therapy (Fig. 5a). The mean fluorescence intensity in laser only group and P-IDI group was fourfold and eightfold higher than control group. Noticeably, with the treatment of P-IDI plus laser, CRT-positive mean fluorescence intensity increased to 24-fold compared to that of PBS group (Additional file 1: Figure S13). The P-IDI group showed 75 nM of ATP secretion in the cell culture medium which was threefold higher than that of PBS group. Upon laser irradiation, ATP secretion in the P-IDI plus laser group increased to 168 nM, which was threefold higher than that of P-IDI group (Fig. 5b). Furthermore, HMGB1 level increased to 164 pg/mL after P-IDI triggered PTT therapy, which was threefold higher than that of other groups (Fig. 5c). Western blot for evaluation the release of DAMPs markers had also been conducted (Additional file 1: Figure S14).

Previous investigations have shown that PTT could trigger ICD of tumor cells to induce the maturation of dendritic cells (DCs) and enhanced therapeutic responses [42]. Then, 4T1 cells treated with PBS, 1064 nm laser irradiation, P-IDI or P-IDI plus laser was incubated with DCs. Then, the maturation of DCs was determined by cytokines ELISA in order to further confirm that P-IDI mediated PTT induces ICD in vivo. With P-IDI plus laser irradiation, DCs released more pro-inflammatory cytokines including 523 pg/mL tumor necrosis factor α (TNF-α) and 239 pg/mL interleukin 6 (IL-6), which was 2.5-fold higher and 1.2-fold than that of PBS group (Fig. 5d, e). Meanwhile, P-IDI plus laser irradiation group showed reduced anti-inflammatory cytokine (interleukin 10 (IL-10) release (239 pg/mL), 1.5-fold lower than that of PBS group (Fig. 5f). Based on all these statistically significant results, the successful ICD effect was verified after PTT treatment.

Through the analysis of splenic lymphocytes of mice in different treatment groups, DCs from P-IDI plus laser group expressed significantly increased CD80^+^CD86^+^ (9.2%), which was threefold higher than that of other groups indicating the maturation of DC cells (Fig. 5g). P-IDI plus laser group also showed 34.72% CD8^+^ cytotoxic T lymphocytes (CTLs) recruitment, which was sevenfold higher than that of other groups indicating the effective activation of innate immune response (Fig. 5h).While the tumors after phototherapy were quickly ablated [43–45], we followed previous reports to finish the immuno-fluorescence imaging of tumor sections, which confirmed that the tumors contained a large number of infiltrating CD8^+^ and CD4^+^ T cells after the treatment with P-IDI plus laser (Additional file 1: Figure S15). Altogether, P-IDI mediated PTT could elicit ICD and lead to the release of DAMPs to induce maturation of DCs, which would provoke subsequent immune responses.

## Conclusions

In summary, we introduced a soluble NIR-II-absorbing diimmonium salt agent with strong absorption at 1000–1100 nm. Investigations in vitro and in vivo demonstrated that they could serve not only as photoacoustic imaging agents but also as thermal therapeutic agents to achieve effective deep-seated cancer treatment with minor side effects on normal tissues. Furthermore, tumor ICD was stimulated to produce tumor antigens and endogenous adjuvants, which lead to release of DAMPs to induce maturation of DCs and recruitment of T cells. This study offered a promising soluble organic NIR-II-absorption agent for photo-theranostic cancer management.

## Supplementary Information

**Additional file 1: Figure S1.** ^1^HNMR spectra of IDI in CDCl_3_ (400 MHz). **Figure S2.** ^13^CNMR spectra of IDI in CDCl_3_ (100 MHz). **Figure S3.** MS spectra of IDI in the positive ESI mode. m/z calcd for C_62_H_92_N_6_^+^ [M]^+^: 920.74; found, 920.74; calcd for C_62_H_92_N_62_^+^ [M]^2+^: 460.37; found, 460.37. **Figure S4.** FT-IR spectra of IPA (black) and IDI (red).** Figure S5.** Magnetic hysteresis curve recorded at room temperature of IDI. **Figure S6.** The stability of IDI and P-IDI in PBS and FBS. **Figure S7.** In vitro cytotoxicity of 4T1 tumor cells incubated with P-IDI (100 μg IDI/mL) under different 1064 nm laser continuous power after 24 h incubation. **Figure S8**. Fluorescence images of Calcein-AM (green fluorescence for live cells) and PI (red fluorescence for dead cells) co-stained tumor cells incubated with P-IDI (100 μg IDI/mL) under different 1064 nm laser continuous power after 24 h incubation (Scale bar: 100 µm). **Figure S9.** (a) Infrared thermal images of tumor-bearing mice illuminated by 1064 nm laser after intravenously treated with P-IDI (10 mg IDI/kg) for different times and power (0.25,0.5,0.75,1 W/cm^2^). (b) Temperature changes of P-IDI upon the laser irradiation from (a). **Figure S10.** (a) In vitro fluorescence images of the organs harvested in BALB/C tumor-bearing mice before and after 12 h post-injection of P-IDI (10 mg IDI/kg). (b) Quantification of mean fluorescence intensity (MFI) of organs in (a). **Figure S11.** Body weight curves of 4T1 tumor-bearing mice after different treatments. **Figure S12.** H&E staining of main organs of mice after different treatments (Scale bar: 100 µm). **Figure S13.** MFI of CRT in Figure 5a. **Figure** **S14**. Western blot determination of HMGB1 and CRT expression under different conditions. **Figure S15.** Staining CD8^+^ and CD4^+^ T cells in tumors (Scale bar: 20 μm). Statistical analysis was assessed via unpaired two-sided Student t-test. *P < 0.05, **P < 0.01, ***P < 0.001, ****P < 0.0001 versus control. **Table S1. **Serum chemistry of mice after intravenous injection with P-IDI (10 mg IDI/kg). Data are mean ± s.d. **Table S2.** Complete blood count of mice after intravenous injection with P-IDI (10 mg IDI/kg). Data are mean ± s.d.

## Data Availability

The datasets used and analyzed during the current study are available from the corresponding author on reasonable request.
