# Correction: NIR-II-absorbing diimmonium polymer agent achieves excellent photothermal therapy with induction of tumor immunogenic cell death

**DOI:** 10.1186/s12951-023-01915-1

**Published:** 2023-06-07

**Authors:** Han Xu, Huaping Deng, Xiaoqian Ma, Yushuo Feng, Ruizhen Jia, Yiru Wang, Yaqing Liu, Wenli Li, Shanshan Meng, Hongmin Chen

**Affiliations:** 1 State Key Laboratory of Molecular Vaccinology and Molecular, Diagnostics & Center for Molecular Imaging and Translational Medicine, School of Public Health, Xiamen University, Xiamen, 361102 China; 2 State Key Laboratory of Organic Electronics and Information Displays & Institute of Advanced Materials (IAM), Jiangsu Key Laboratory for Biosensors, Nanjing University of Posts & Telecommunications, Nanjing, 210023 People’s Republic of China

**Correction** **:** **Journal of Nanobiotechnology (2023) 21:132** **https://doi.org/10.1186/s12951-023-01882-7**

Following publication of the original article [1], the authors reported that assignment of affiliation 1 to author Hongmin Chen was omitted.

The affiliation assignment has been corrected above and the original article [1] has been updated.
