# Supplementary material for: NIR-II-absorbing diimmonium polymer agent achieves excellent photothermal therapy with induction of tumor immunogenic cell death
Source: J Nanobiotechnology. 2023 Apr 20;21:132. doi: 10.1186/s12951-023-01882-7 (PMC10116819; doi:10.1186/s12951-023-01882-7)
Supplement: Supplementary file 1 — Additional file 1: Figure S1. 1HNMR spectra of IDI in CDCl3 (400 MHz). Figure S2. 13CNMR spectra of IDI in CDCl3 (100 MHz). Figure S3. MS spectra of IDI in the positive ESI mode. m/z calcd for C62H92N6+ [M]+: 920.74; found, 920.74; calcd for C62H92N62+ [M]2+: 460.37; found, 460.37. Figure S4. FT-IR spectra of IPA (black) and IDI (red). Figure S5. Magnetic hysteresis curve recorded at room temperature of IDI. Figure S6. The stability of IDI and P-IDI in PBS and FBS. Figure S7. In vitro cytotoxicity of 4T1 tumor cells incubated with P-IDI (100 μg IDI/mL) under different 1064 nm laser continuous power after 24 h incubation. Figure S8. Fluorescence images of Calcein-AM (green fluorescence for live cells) and PI (red fluorescence for dead cells) co-stained tumor cells incubated with P-IDI (100 μg IDI/mL) under different 1064 nm laser continuous power after 24 h incubation (Scale bar: 100 µm). Figure S9. (a) Infrared thermal images of tumor-bearing mice illuminated by 1064 nm laser after intravenously treated with P-IDI (10 mg IDI/kg) for different times and power (0.25,0.5,0.75,1 W/cm2). (b) Temperature changes of P-IDI upon the laser irradiation from (a). Figure S10. (a) In vitro fluorescence images of the organs harvested in BALB/C tumor-bearing mice before and after 12 h post-injection of P-IDI (10 mg IDI/kg). (b) Quantification of mean fluorescence intensity (MFI) of organs in (a). Figure S11. Body weight curves of 4T1 tumor-bearing mice after different treatments. Figure S12. H&E staining of main organs of mice after different treatments (Scale bar: 100 µm). Figure S13. MFI of CRT in Figure 5a. Figure S14. Western blot determination of HMGB1 and CRT expression under different conditions. Figure S15. Staining CD8+ and CD4+ T cells in tumors (Scale bar: 20 μm). Statistical analysis was assessed via unpaired two-sided Student t-test. *P < 0.05, **P < 0.01, ***P < 0.001, ****P < 0.0001 versus control. Table S1. Serum chemistry of mice after intravenous injection with P- [file 12951_2023_1882_MOESM1_ESM.docx]

**Support information**

**NIR-II-Absorbing Diimmonium Polymer Agent Achieves Excellent Photothermal Therapy with Induction of Tumor Immunogenic Cell Death**

Han Xu, Huaping Deng, Xiaoqian Ma, Yushuo Feng, Ruizhen Jia, Yiru Wang, Yaqing Liu, Wenli Li, Shanshan Meng, Hongmin Chen*

H. Xu, H. Deng, X. Ma, Y. Feng, R. Jia, Y. Wang, Y. Liu, W. Li, S. Meng, Prof. H. Chen

State Key Laboratory of Molecular Vaccinology and Molecular Diagnostics & Center for Molecular Imaging and Translational Medicine, School of Public Health, Xiamen University, Xiamen 361102, China

* E-mail: [hchen@xmu.edu.cn](mailto:hchen@xmu.edu.cn)

Prof. H. Chen

State Key Laboratory of Organic Electronics and Information Displays & Institute of Advanced Materials (IAM), Jiangsu Key Laboratory for Biosensors, Nanjing University of Posts & Telecommunications, Nanjing 210023, P. R. China

**Experimental section**

**Synthesis of IDI**

N, N, N′, N′-Tetrakis[4-(diisobutylamino)phenyl]-1,4- phenylenediamine (IPA) (0.5 g, 0.54 mmol) and lithium bis(oxalate) borate (0.25 g, 1.29 mmol) were added to a two-necked flask. Then, 2.5 g of dichloromethane and 1.0 g of ethanol were added, and the mixture was refluxed for 2 h. When the solution turns dark green after 2 h, sodium persulfate (0.175 g, 0.765 mmol) and 4.0 g of water were added into the mixture and refluxed for 2 h. Then, the reaction mixture was terminated by adding large amounts of water and diluted with water and extracted by dichloromethane for several times. The combined organic phase was dried with anhydrous MgSO_4_. After the removal of the solvent to produce IDI as a black solid (467 mg, 89.42%).

NMR Characterization of IDI: The NMR peaks of the diimmonium compounds are broad without any assignable peaks.

HPLC/MS m/z (ESI) calcd for C_62_H_92_N_6_^+^ [M]^+^ 920.74; found, 920.74; calcd for C_62_H_92_N_6_^2+^ [M]^2+^ 460.37; found, 460.37.

**Magnetic properties measurement**

The magnetic properties of IDI were performed on a sample of arbitrary shape with mass of 10.1 mg, and measured using physical property measurement system (PPMS; Quantum Design, USA), in which the magnetic field intensity was measured at room temperature and ±2 T.

**Preparation of P-IDI nanoparticles**

The nanoprecipitation method was adopted for the preparation of the drug-loaded nanocarriers [1]. Briefly, 1 mg IDI was dissolved in 1 mL THF before adding to 10 mL deionized water containing 7 mg DSPE-PEG-NH2. The mixture was allowed to react overnight at room temperature, under moderate magnetic stirring. P-IDI nanocomposites were purified by centrifugation and washed 3 times with an ultrafiltration. The final product was suspended in 1 mL of distilled water as stock solution for further use.

**Photothermal performance measurement**

Photothermal effect was monitored with a FLIR A×5 camera (FLIR Systems Inc., Wilsonville, OR, USA) when 0.1 mL samples of P-IDI (100 μg IDI/mL) were continuously exposed under laser irradiation (808 nm, 1 W/cm^2^ and 1064 nm, 1 W/cm^2^) for 300 s.

The photothermal conversion efficiency of P-IDI was calculated by monitoring the temperature change of P-IDI in aqueous dispersion as a function of time under continuous laser irradiation. When the temperature reached to a plateau, the laser was turned off and the temperature was recorded during the cooling stage until the temperature decreased to the room temperature.

The photothermal conversion efficiency (η) was calculated using the following reported equation[2]

$$\sum_{i} m_{i}C_{p,i}\frac{ⅆT}{ⅆt}=Q_{s}-Q_{loss}$$

Where *m_i_* was the mass and *C_p,i_* was the heat capacity of system components, respectively. *Q_s_* was the photothermal heat energy input by irradiating P-IDI solution with NIR laser, and *Q_loss_* was thermal energy lost to the surroundings. When the temperature was the maximum, the system was in balance.

$$Q_{s}=Q_{loss}=hS_{\Delta}T_{max}$$

Where *h* was heat transfer coefficient, *S* was the surface area of the container, and Δ*T_max_* was the maximum temperature change.

$$\eta=\frac{hS_{\Delta}T_{max}}{I(1-{10}^{-A\lambda})}$$

Where *I* was the laser power and *λ* was the absorbance of P-IDI solution at the wavelength of 1064 or 808 nm.

In order to get the *hS*, a dimensionless driving force temperature, *θ* was introduced as follows:

$$\theta=\frac{T-T_{Surr}}{T_{max}-T_{surr}}$$

Where *T* was the temperature of P-IDI solution, *T_max_* was the maximum system temperature, and *T_surr_* was the initial temperature.

$$\tau_{s}=\frac{\Sigma_{i}m_{i}c_{p,i}}{hS}$$

A sample system time constant *τ_s_*, thus

$$\frac{ⅆ\theta}{ⅆt}=\frac{l}{\tau_{s}}\frac{Q_{s}}{hS\Delta T_{max}}-\frac{\theta}{\tau_{s}}$$

When the laser was off, Q_s_ = 0, ⅆθ/ⅆt = -θ/τ_s_, *t* = -τ_s_ *ln*θ. So, *hS* could be calculated from the slope of cooling time *vs* *lnθ*. The time constant (*τ_s_*) of heat transfer from P-IDI was determined to be 89.68 s and 73.67 s for 808 nm and 1064 nm, respectively. The ∆*Tmax* of P-IDI was 19.65 °C and 35.24 °C for 808 nm and 1064 nm laser irradiation, respectively. Therefore, the photothermal conversion efficiency (η) of P-IDI was calculated to be 29.8% and 34.7% at 808 nm and 1064 nm, respectively.

**Hemolysis assay**

The Red blood cells (RBCs) were isolated from serum by centrifugation of the mixture containing 0.5 mL blood sample and 1 mL PBS solution at 4500 rpm for 3.5 min. PBS was used to wash the RBCs five times and dilute the purified cells to 5 mL. Then certain volume of diluted RBCs suspension (0.3 mL) was added to quadruple volume of PBS solution with different concentrations of P-IDI (25-100 μg IDI/mL). The mixtures were vortexed and kept to stand for 3 h at room temperature. Samples were then centrifuged to measure the absorbance of the supernatants at 541 nm by an UV–vis spectroscopy. RBCs treated with deionized water and PBS were set as positive and negative controls.

**Cellular uptake efficiency and degradation studies**

4T1 cells were incubated with Cy5.5-labeled P-IDI (100 μg IDI/mL) for 0.5, 1, 2, 4 and 6 h, respectively. After washed by PBS for twice, 4T1 cells were stained with Hochest33342. Subsequently, the treated cells were imaged by CLSM (excitation wavelengths: 405 nm for Hochest33342 and 585 nm for Cy5.5, respectively). Besides, 4T1 cells co-incubated with P-IDI (100 μg IDI/mL) for 2 and 24 h were collected and fixed overnight with glutaraldehyde (2.5%, w/w) at 4^o^C. Then all samples were made bio-transmission electron microscopy (Bio-TEM) specimens with standard procedures for observation.

**Cytotoxicity**

4T1 cells were seeded in a 96-well plate at a density of 1×10^4^ cells/100 μL and incubated for 24 h in DMEM medium supplemented with 10 % of fetal bovine serum (FBS), 100 units/mL of penicillin and 100 mg/mL of streptomycin at 37 °C in a humidified atmosphere of 5 % CO_2_. Then the cells were treated with P-IDI at different concentrations and co-incubated for another 24 h. 5.0 mg/mL stock solution of MTT was prepared in PBS and this stock solution (10 μL) was added to each well. After additional 4.0 h incubation, the medium and MTT were removed, and the MTT-formazan crystals in each well were dissolved in 150 μL of DMSO. The absorbance of the suspension was recorded by a microplate reader at wavelength of 490 and 570 nm.

The phototoxicity of P-IDI was also evaluated. The 4T1 cells cultured in 96-well plates as described above were incubated with P-IDI at different concentrations (12, 25, 50, 100 and 200 µg IDI/mL) for 6 h. Then the cells were irradiated by a 1064 nm NIR laser at a power density of 1 W/cm^2^ for 5 min. The area of each well was fully covered by the laser spot. After being illuminated, the cells were incubated for another 24 h before the MTT assay. The cell viability was normalized to the control group without any treatment.

To visualize the live/dead cells, 4T1 cells were treated with P-IDI (100 μg IDI/mL) for 12 h and subsequently exposed to laser irradiation (1064 nm, 1 W cm^−2^) for 5 min. After incubation for 4 h, the cells were stained with calcein-AM/PI for 30 min. Finally, the labeled cells were rinsed twice with PBS and imaged by CLSM (excitation wavelengths: 488 nm and 530 nm for calcein-AM and PI, respectively).

**Detections of immunogenic cell death (ICD) biomarkers**

4T1 cells were seeded in a confocal dish at a density of 1×10^5^ cells/100 μL and incubated overnight in DMEM medium, then cells were incubated with PBS, Laser irradiation, P-IDI or P-IDI + Laser irradiation. After incubation for another 16 h, cells were washed and stained by anti-CRT antibody (Alexa Fluor® 647) for 30 min. After washing, the expression of CRT was detected by confocal microscopy. To detect the HMGB1 and ATP release, 4T1 cells were treated by the same method above, after incubation, the cell culture medium was collected and used for the detection of HMGB1 and ATP. The HMGB1 evaluation was conducted by an Elisa assay. The ATP detection was conducted by an ATP detection kit according to the manufacture’s instruction.

4T1 cells were seeded in 6-well plates at a density of 1×10^6^cells/100 μL and incubated overnight in DMEM medium, then cells were treated with PBS, Laser irradiation, P-IDI or P-IDI + Laser irradiation, respectively. At 4 h post-treatment, the cells were lysed in a lysis buffer. Proteins were separated by 12% tris-Gly SDS-PAGE, then transferred to polyvinylidene difluoride membrane, and immunoblotted with primary antibodies against GAPDH, HMGB1 and CRT. Subsequently, HRP-conjugated secondary antibody was applied. The protein bands were visualized with Spakjade ECL super using a Bio-Rad imaging system.

Bone marrow–derived DCs were harvested from BALB/c mice by the established method[3]. 2×10^5^ DCs were seed in a 24-well culture plate per well for 12 h. Then the cells were incubated with 4T1 cells (2×10^5^) after different treatment. After incubation for 24 h, supernatants of DCs were collected by centrifuging 10 min (2000 r/min). Cytokine secreted from cells were detected by TNF-α, IL-6 and IL-10 ELISA kits (Elabscience), following the manufacturer’s instructions.

**Animal experiments**

Our animal experiments were conducted on female BALB/c mice, which were purchased from Shanghai SLAC Laboratory Animal Co. Ltd (Shanghai, China). All the experiments were under the guidelines of the Regional Ethics Committee for Animal Experiments and the Care Regulations approved by the Institutional Animal Care and Use Committee of Xiamen University. 4T1 tumor-bearing mice model were established by subcutaneously injecting of 4T1 cells (2*10^6^) suspended 100 μL into the back of the hind leg.

**Serum chemistry and hematological analysis**

To verify the biosafety, BALB/c mice were intravenously injected with P-IDI (10 mg IDI/kg). Blood samples were collected from the mice eye socket vein for serum chemistry and complete blood analysis at day 7 and 14 after injection (n = 3). The results were compared with bloods from the same mice before injection. The blood serum samples were analyzed using an Auto Biochemistry Analyzer (Mindray, BS-220) for some liver and renal function markers: alanine transaminase (ALT), aspartate transaminase (AST), creatinine (CREA) and blood urea nitrogen (UREA). For complete blood analysis, a series of indicators like white blood cell, red blood cell, hemoglobin, etc., were measured using an Auto Hematology Analyzer (Mindray, BC-2600).

***In vitro* and *in vivo* imaging**

The *in vitro* PA signals of P-IDI solution (0, 60, 120, 250, 500, and 1000 μg IDI/mL) at various concentrations were collected with the excitation wavelengths at 808 nm. To investigate *in vivo* PA imaging after intravenous administration with P-IDI (10 mg IDI/kg), the *in vivo* PA images and signal intensities at tumor sites of the mice were monitored at various time intervals post injection. PA imaging *in vitro* and *in vivo* were all accomplished by a Vevo LAZR-X system with the following parameter: Frequency: 60 MHz; 2D gain: 0 dB; PA gain:30 dB; excitation wavelength: 808 nm.

***In Vivo* PTT effect**

The tumor volume of each mouse was kept monitoring with a vernier caliper every 3 days and calculated according to the formula: Volume = (tumor length) × (tumor width)^2^/2. Relative tumor volumes were all normalized to their initial sizes. When the tumor size reached about 100 mm^3^, mice of treatment group (n = 4) were intravenously injected 200 μL of PBS or P-IDI (10 mg IDI/kg), respectively. The tumor regions of mice were irradiated with a NIR laser for 5 min (1064 nm, 1 W/cm^2^). Mice of control group were kept in the same circumstance without any treatment. Mice were euthanized on day 21 and the tumors were weighted. Major organs such as heart, liver, lung, spleen, kidney were excised and resected for hematoxylin and eosin (H&E) staining to appraise the in vivo biocompatibility of P-IDI. H&E staining was also applied in tumor tissue sections of control and treatment groups 24 h after irradiation (1064 nm, 1 W/cm^2^, 5 min).

***In vitro* analysis of different groups of T cells and dendritic cells (DCs)**

To analyze immune cells by flow cytometry, spleens of mice after various treatments were collected and stained according to the manufacturer’s protocols. In brief, to analyze memory T cells, cells from spleens were stained with antibodies against CD8a-APC (BioLegend, catalog no. 100712) and CD3-PE (BioLegend, catalog no. 100206). To analyze the maturation of DCs, cells from spleens were stained with antibodies against CD80-APC (BioLegend, catalog no. 104714), CD86-PE (BioLegend, catalog no. 159204), CD11c-FITC (BioLegend, catalog no. 117306).


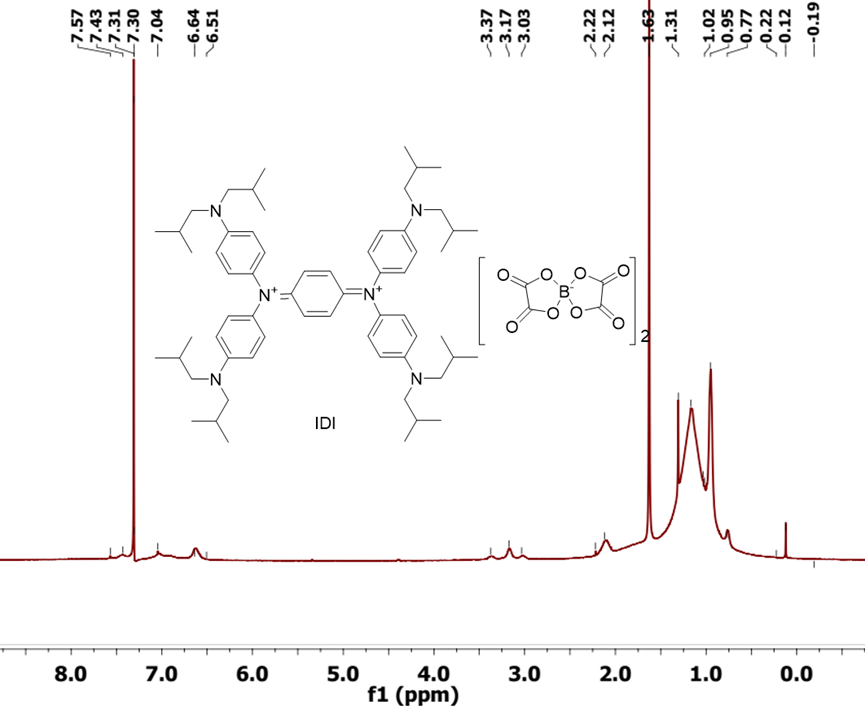


**Figure S1**. ^1^HNMR spectra of IDI in CDCl_3_ (400 MHz).


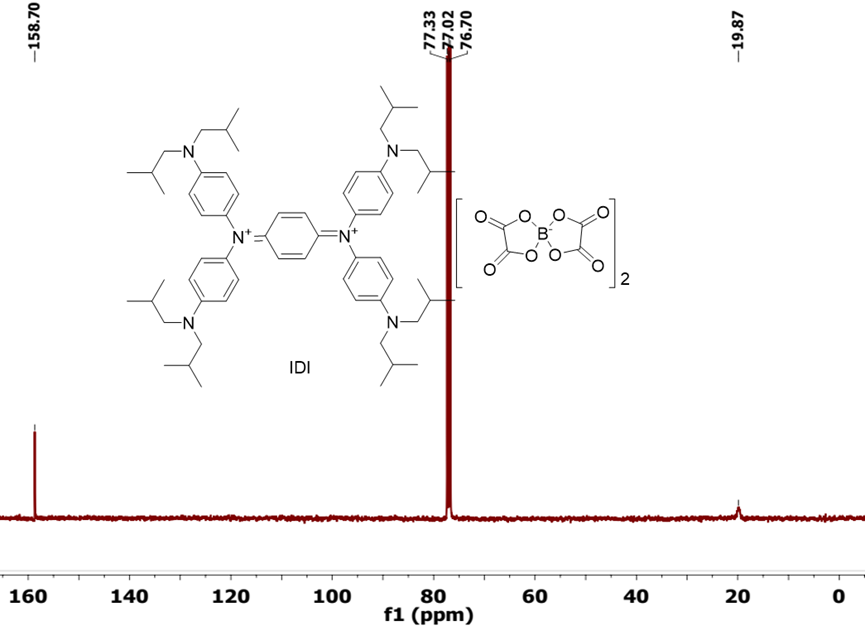


**Figure S2**. ^13^CNMR spectra of IDI in CDCl_3_ (100 MHz).


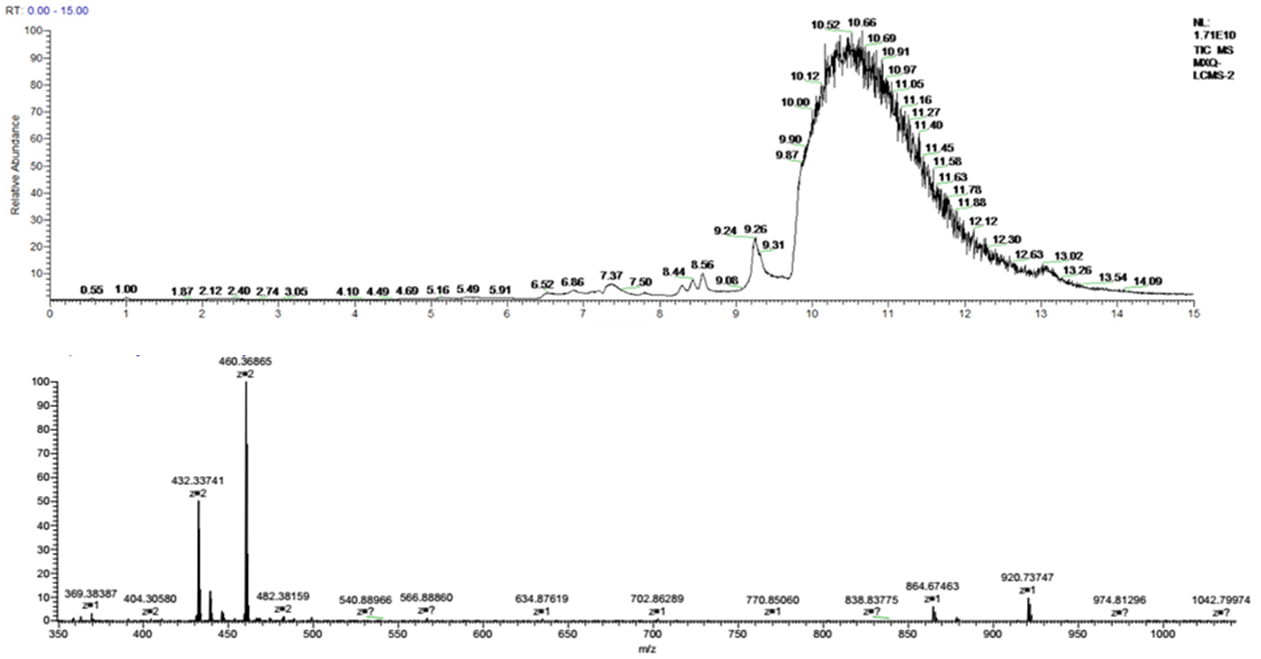
**Figure S3**. MS spectra of IDI in the positive ESI mode. m/z calcd for C_62_H_92_N_6_^+^ [M]^+^: 920.74; found, 920.74; calcd for C_62_H_92_N_62_^+^ [M]^2+^: 460.37; found, 460.37.


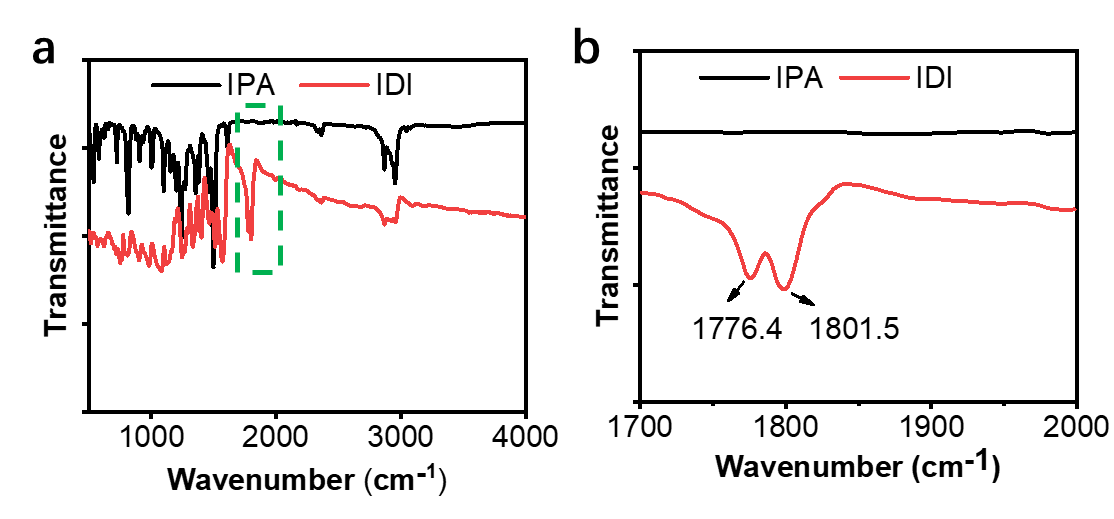


**Figure S4**. FT-IR spectra of IPA (black) and IDI (red).


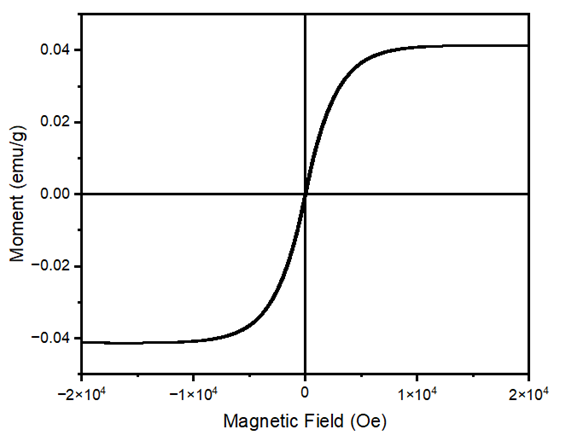


**Figure S5.** Magnetic hysteresis curve recorded at room temperature of IDI.

*
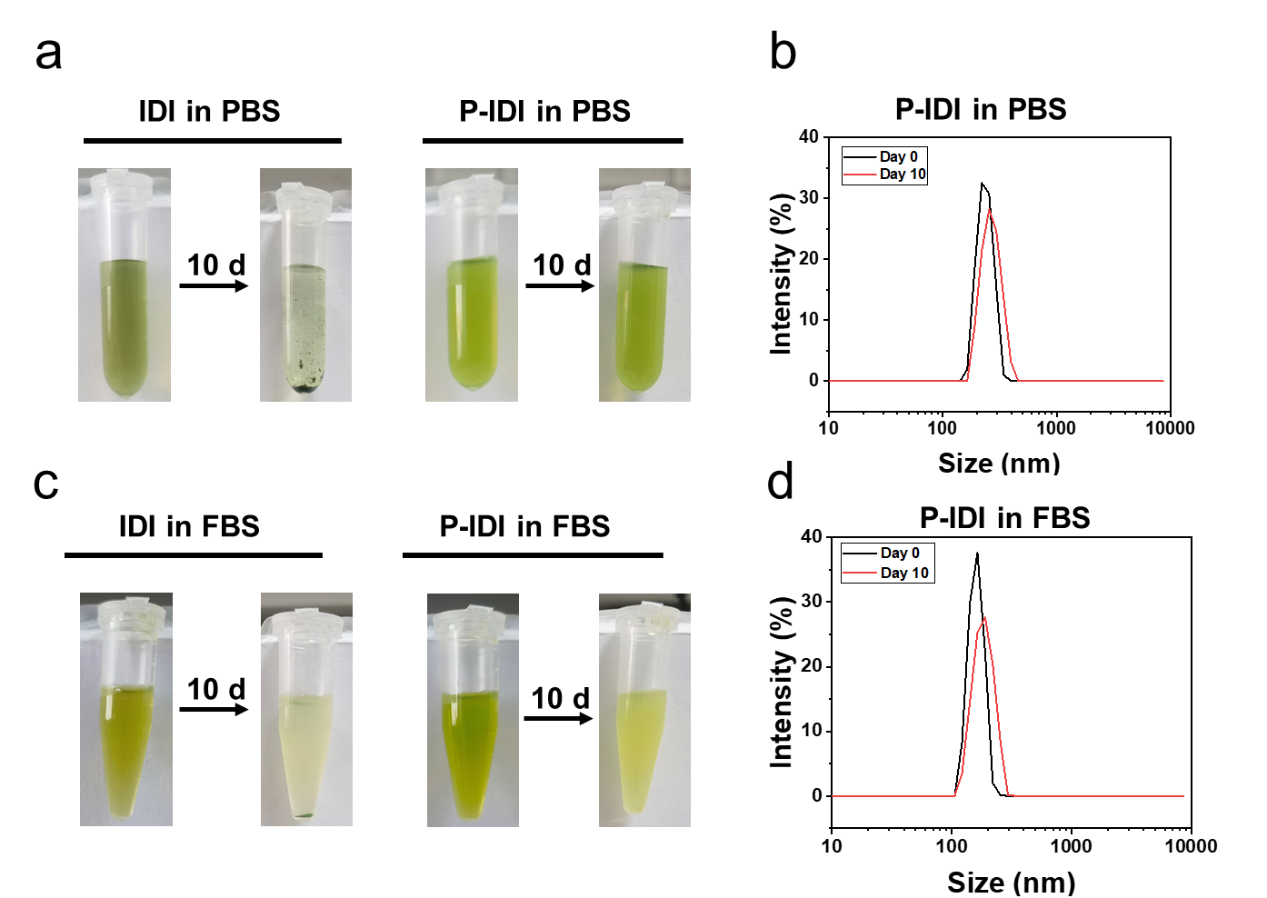
*

**Figure S6**. The stability evaluation. (a) Images of IDI and P-IDI diffusing in PBS for ten days. (b) Comparison of the hydration particle sizes of P-IDI in PBS at day 0 and day 10. (c) Images of IDI and P-IDI diffusing in FBS for ten days. (d) Comparison of the hydration particle sizes of P-IDI in FBS at day 0 and day 10.

**Figure S7**. In vitro cytotoxicity of 4T1 tumor cells incubated with P-IDI (100 μg IDI/mL) under different 1064 nm laser continuous power after 24 h incubation**.**
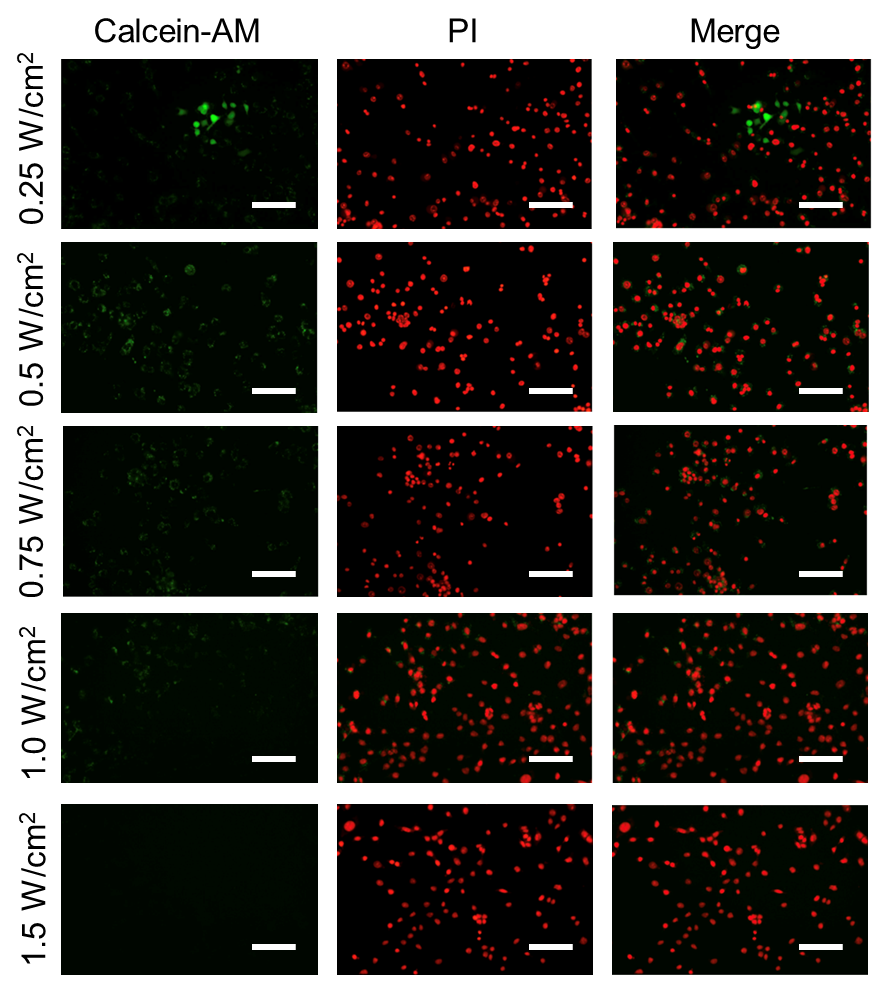


**Figure S8**. Fluorescence images of Calcein-AM (green fluorescence for live cells) and PI (red fluorescence for dead cells) co-stained tumor cells incubated with P-IDI (100 μg IDI/mL) under different 1064 nm laser continuous power after 24 h incubation (Scale bar 100 µm).


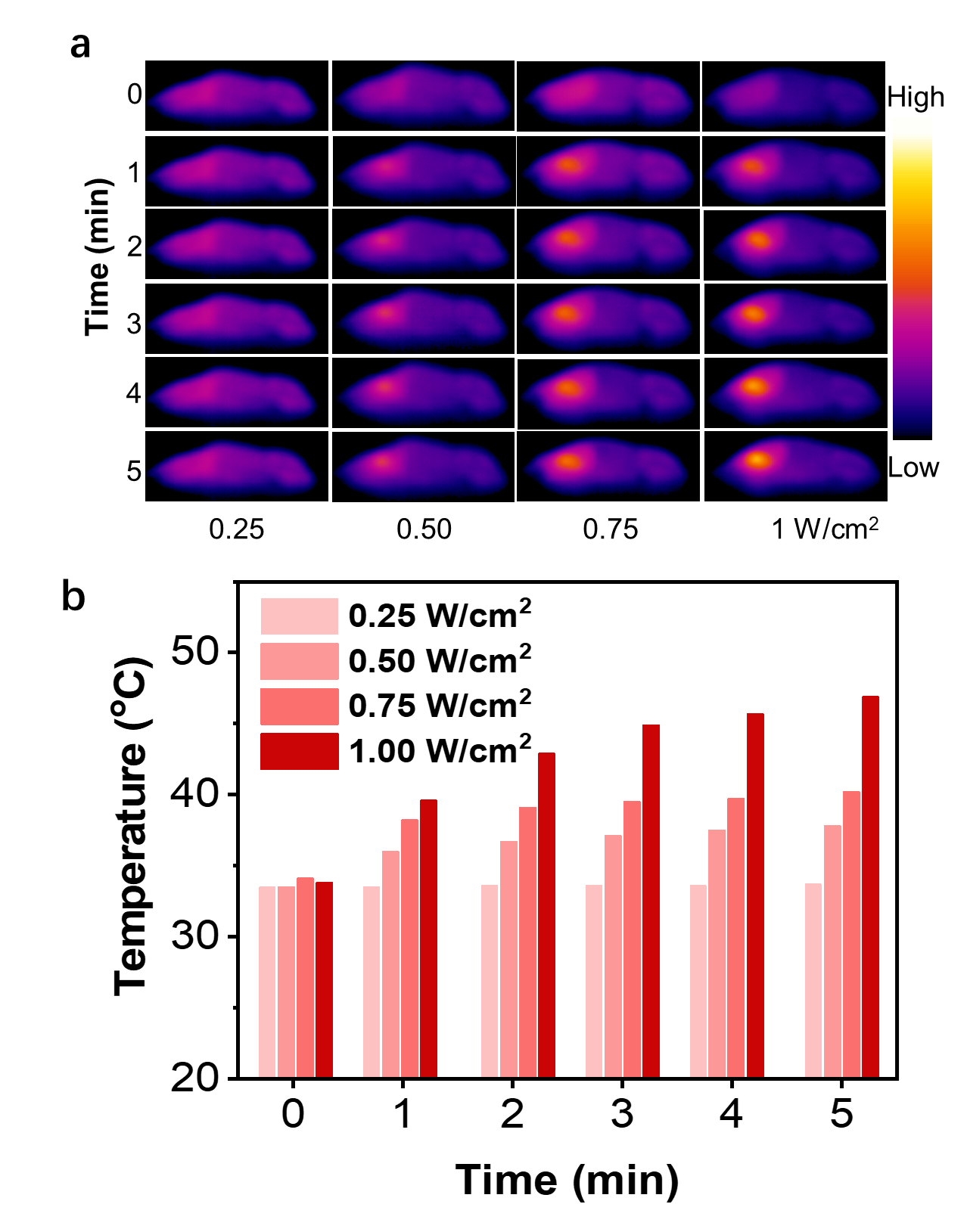


**Figure S9.** (a) Infrared thermal images of tumor-bearing mice illuminated by 1064 nm laser after intravenously treated with P-IDI (10 mg IDI/kg) for different times and power (0.25, 0.5, 0.75, 1 W/cm^2^). (b) Temperature changes of P-IDI upon the laser irradiation from (a).


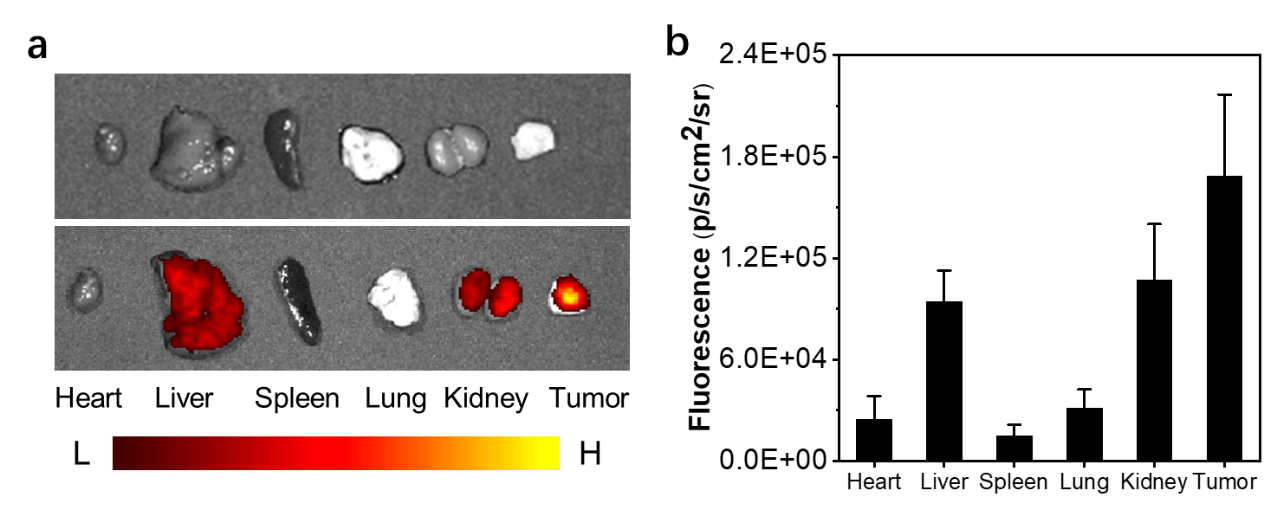


**Figure S10.** (a) In vitro fluorescence images of the organs harvested in BALB/C tumor-bearing mice before and after 12 h post-injection of P-IDI (10 mg IDI/kg). (b) Quantification of mean fluorescence intensity (MFI) of organs in (a).

**Figure S11.** Body weight curves of 4T1 tumor-bearing mice after different treatments.


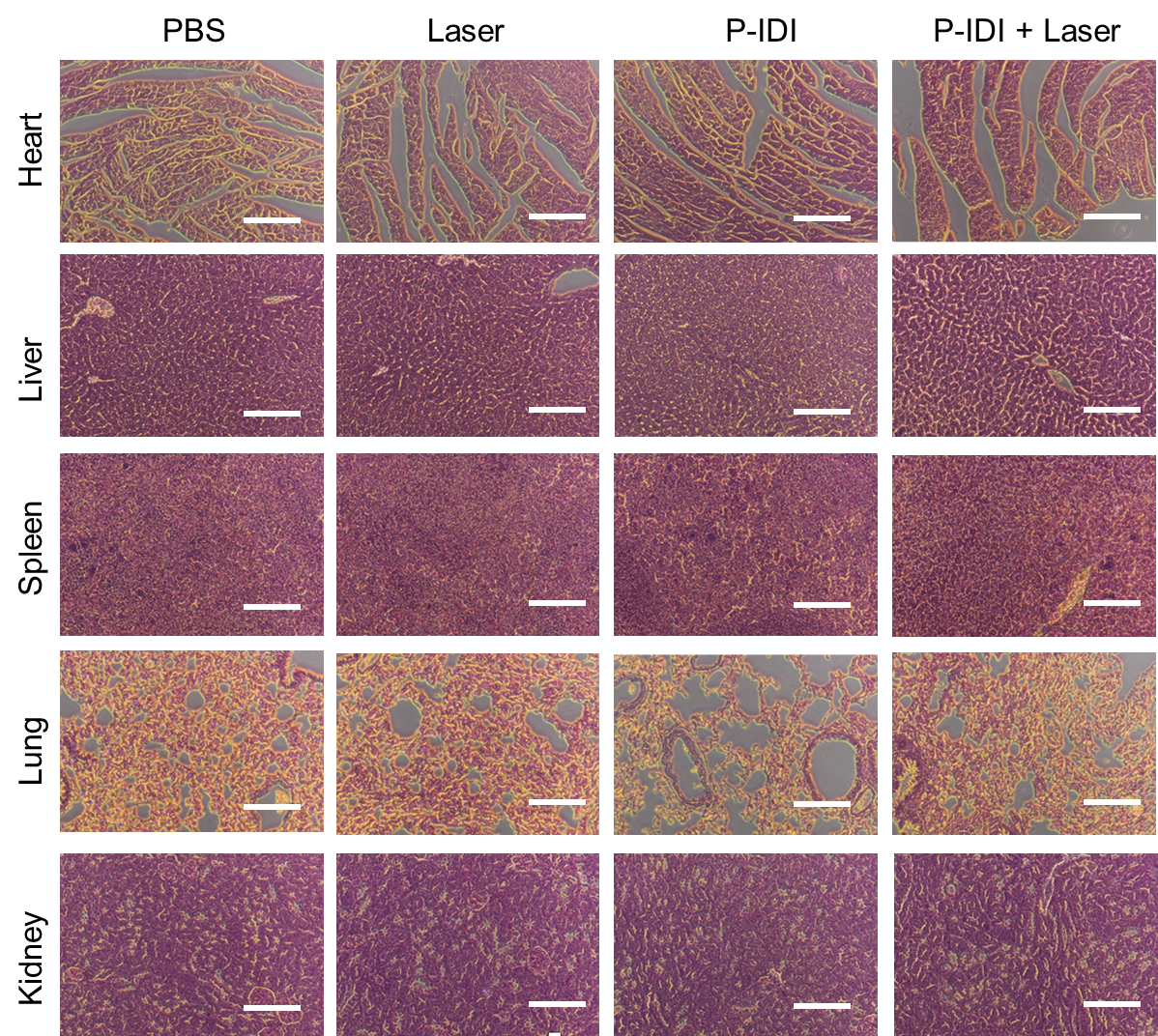


**Figure S12.** H&E staining of main organs of mice after different treatments (Scale bar: 100 µm).

**Figure S13.** Quantification of mean fluorescence intensity (MFI) of CRT in Figure 5a. Statistical significance was assessed via unpaired two-sided Student t-test. *P < 0.05, **P < 0.01, ***P < 0.001, ****P < 0.0001 versus control.


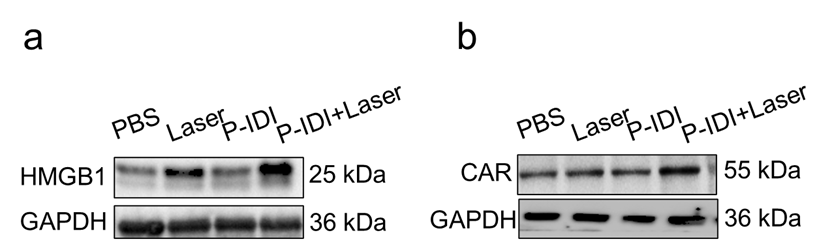


**Figure S14**. Western blot determination of HMGB1 and CRT expression under different conditions.


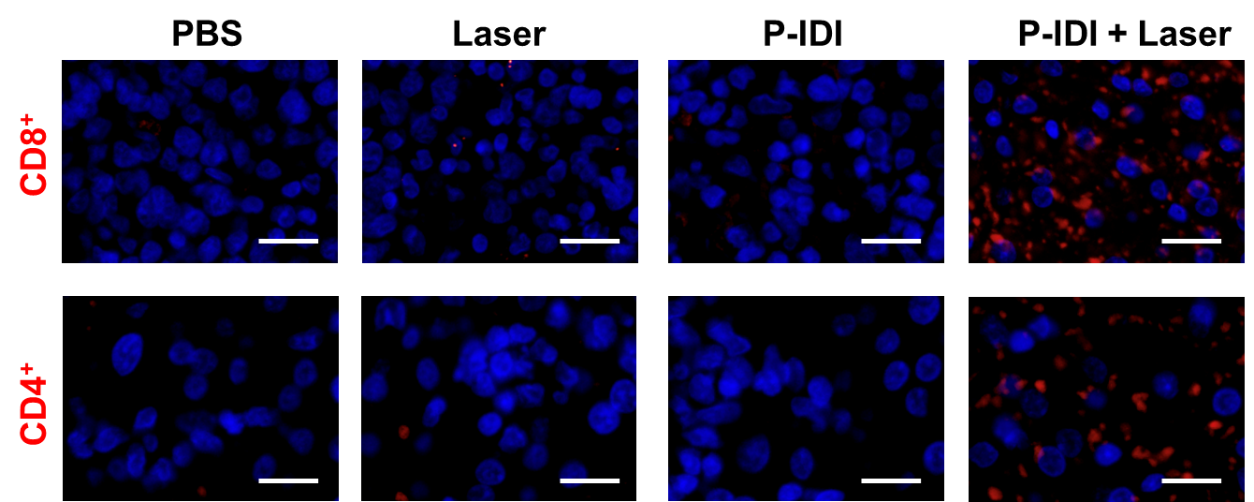


**Figure S15.** Staining CD8^+^ and CD4^+^ T cells in tumors (Scale bar = 20 μm).

**Table S1.** Serum chemistry of mice after intravenous injection with P-IDI (10 mg IDI/kg). Data are mean ± s.d.

**Table S2.** Complete blood count of mice after intravenous injection with P-IDI (10 mg IDI/kg). Data are mean ± s.d.

**References**

1. Feng L, Zhu C, Yuan H, Liu L, Lv F, Wang S: **Conjugated polymernanoparticles: preparation, properties, functionalization and biological applications.** *Chemical Society Reviews* 2013, **42:**6620-6633.

2. Xiang H, Zhao L, Yu L, Chen H, Wei C, Chen Y, Zhao Y: **Self-assembled organic nanomedicine enables ultrastable photo-to-heat converting theranostics in the second near-infrared biowindow.** *Nature Communications* 2021, **12:**218.

3. Yan D, Wang M, Wu Q, Niu N, Li M, Song R, Rao J, Kang M, Zhang Z, Zhou F, et al: **Multimodal Imaging-Guided Photothermal Immunotherapy Based on a Versatile NIR-II Aggregation-Induced Emission Luminogen.** *Angewandte Chemie International Edition* 2022, **61:**e202202614.
